# What Does COVID‐19 Look Like? A ‘Draw, Write and Tell’ Exploration of Children's Postpandemic Understanding of COVID‐19

**DOI:** 10.1111/cch.70374

**Published:** 2026-11-06

**Authors:** Rea Evangelia Michalopoulou, Freya Murray, Shuran Zhang, Benjamin Bach, Panagiotis Kourtesis, Joanne M. Williams

**Affiliations:** ^1^ Department of Clinical and Health Psychology, School of Health in Social Science The University of Edinburgh Edinburgh UK; ^2^ Bivwac Team French National Institute for Research in Digital Science and Technology (Inria) Bordeaux France; ^3^ Design Informatics, School of Informatics University of Edinburgh Edinburgh UK; ^4^ Department of Psychology The American College of Greece Athens Greece; ^5^ Department of Psychology National and Kapodistrian University of Athens Athens Greece; ^6^ Department of Informatics and Telecommunication of the National and Kapodistrian University of Athens Athens Greece; ^7^ Department of Psychology The University of Edinburgh Edinburgh UK

**Keywords:** children, common sense model (CSM), COVID‐19, drawings, draw–write–tell (DWT), illness understanding, naïve biology

## Abstract

**Objectives:**

This study explored children's understanding of COVID‐19 after the pandemic using the ‘Draw, Write and Tell’ (DWT) method. It assessed the accuracy of children's drawings based on the features of the Common Sense Model (CSM), examined developmental trends and explored the visual representations children attribute to COVID‐19 in their drawings.

**Design:**

A cross‐sectional participatory design using the DWT method in individual data‐collection sessions was used.

**Methods:**

Seventy children, 5–11 years old, created a picture to depict COVID‐19, using drawings and words. Children were then invited to describe their picture to the researcher. Data were collected individually in schools. The complete visual artefact comprised the child's original drawing, accompanying words and annotations provided by the researcher. The artefacts were analysed using a dual approach of Content Analysis and Reflexive Thematic Analysis.

**Results:**

Older children demonstrated a significantly more accurate representation of COVID‐19. Accuracy was the highest in the CSM‐informed dimensions of Identity, Severity and Cause, while understanding of Timeline, Curability and Treatment remained consistently underdeveloped across all age groups. Two themes surrounding children's understanding of COVID‐19 were constructed: COVID‐19 as an illness and COVID‐19 as a biological entity. Interestingly, children across all ages anthropomorphised COVID‐19, with older children being more likely to do so. Lastly, children discussed their sources of COVID‐19 information.

**Conclusion:**

Children's understanding of COVID‐19 is multifaceted, encompassing both the concept of the illness and its biological causes. This holds broader implications in the postpandemic world for the design and dissemination of developmentally appropriate child‐specific health information campaigns and biology education in the primary school years.

## Key Points

1. Children's COVID‐19 drawings reflect and expand beyond the features of the Common Sense Model of Illness (CSM). The observed uneven understanding of the CSM dimensions highlights specific areas that educational interventions should target.
2. Older children demonstrated a significantly more accurate overall understanding of COVID‐19. These developmental trends can guide the design and dissemination of developmentally appropriate child‐specific health information campaigns and biology education in the primary school years.
3. The Draw–Write–Tell methodology is a useful tool for exploring children's understanding of health and illness, as it provides a comprehensive view of their understanding of COVID‐19. Children's drawings showed that COVID‐19 encapsulates both the concept of the illness and its biological entity.
4. Children across all ages anthropomorphised COVID‐19 in their drawings, with older children being more likely to do so. This invites a discussion of its use in health communication aimed at children.
5. Children did not mention parents or teachers as their sources for COVID‐19 information. Ultimately, these findings highlight the importance of visual media in the dissemination of health information to this age group.

## 1 Introduction

Since the beginning of the pandemic in 2020, COVID‐19 has been an invisible, complex threat that disrupted all aspects of daily life. Given this unprecedented public health emergency, health information campaigns were omnipresent, designed largely by and for adults (Bray, Blake, et al. 2021; Christidou et al. 2021; Rowland and Cook 2021; Thompson et al. 2021). Thus, children were indirect recipients of these messages about SARS‐CoV‐2, its transmission risks and preventative practices. A large‐scale report (Götz et al. 2020) highlighted the impact of this discrepancy, concluding that children were feeling worried while holding some knowledge about COVID‐19 and could benefit from more age‐appropriate information. Since May 2023 (World Health Organisation [WHO] 2023), COVID‐19 no longer constitutes a public health emergency of international concern (PHEIC). However, it provided a unique context for understanding the way children conceptualise emergent infectious diseases, even with the pandemic being ostensibly over. Exploring children's visual representations of COVID‐19, as a route to making their invisible conceptualisation visible, can provide a crucial framework for informing future health messaging designed for children.

### 1.1 Children's Understanding of Health Threats

Developmental trends have been observed in children's understanding of health threats and appear to vary by disease. Children showed a more detailed understanding of AIDS compared to the flu, which has been attributed to exposure to information during the AIDS epidemic (Sigelman 2022). Other illnesses like chickenpox or measles are relatively well understood, with children of all ages demonstrating knowledge about how to prevent and recover from them (Bir and Podmore 1990). As anticipated, the literature exploring children's understanding of COVID‐19 is scarce. Thompson et al. (2021) found that children understand COVID‐19 across three themes: COVID‐19 as a contagious illness, negative emotions around the pandemic and social responsibility. The latter aspect provides an intriguing component to the illness construct, as it does not emerge in the understanding of other diseases. Labotka and Gelman (2023) reported that children's understanding of COVID‐19 and the common cold was largely similar; however, when a difference was present, children held a higher accuracy in their conceptualisation of COVID‐19 compared to the common cold. Critically, the aforementioned data were collected during the pandemic, when information about COVID‐19 was ubiquitous. It remains unknown whether this high level of accuracy in COVID‐19 understanding remained after the pandemic.

Children's understanding of health and illness has been investigated through several theoretical and conceptual perspectives (Brandone 2024; Koopman et al. 2004; Labotka and Gelman 2023; Natapoff 1982). Much of the early research in the area was either atheoretical or informed by Piaget's theory of cognitive development (e.g., Bibace and Walsh 1980; Perrin and Gerrity 1981) and argued against a coherent understanding of illness till late adolescence. In contrast, more recent studies show that children's understanding of illness begins to develop from the preschool years (Kalish 1996; Myant and Williams 2005) and is increasing in accuracy and sophistication with age (Sigelman and Glaser 2019). While the capacity to understand health concepts from a young age is apparent, the extent and precise way development influences children's understanding of illness remains an area of ongoing debate and exploration.

This study adopted the Common Sense Model (CSM) of Illness (Leventhal et al. 1980, 1992, 2016) as a suitable framework to organise the nuance in children's illness perceptions, which is composed of five core dimensions: Identity, Cause, Timeline, Consequences and Control. Although the framework was designed in relation to adults, it has been effective to organise children's representations across a range of different illness conditions and previous epidemics, such as the flu, common cold, AIDS and H1N1 influenza (swine flu) (e.g., Karademas et al. 2013; Law et al. 2014; Myant and Williams 2005; Williams et al. 2012; Wong and Sam 2011). Developmental trends have been observed within the CSM, as older children demonstrate a better understanding of the cause, timeline, curability and consequences dimensions (Fox et al. 2008; Wickering et al. 2023). Relatively little research has been done using the CSM as a framework for COVID‐19 (e.g., Hayes et al. 2022; Möller et al. 2022), and importantly, no application of the CSM has been made in children's understanding of COVID‐19.

### 1.2 Drawings and Children's Health Concepts

Drawings have previously been used as an effective tool to elucidate the CSM dimensions with both children and adults (Broadbent et al. 2019). Draw, Write and Tell (DWT) is a versatile creative research method that asks the participant to provide a drawing based on a prompt, include annotations and describe this creation to the researcher (Angell et al. 2015). It has long been identified as a useful tool to explore children's understanding of health and illness (e.g., Bradding and Horstman 1999; Liamputtong and Fernandes 2015; Pope et al. 2019; Xie et al. 2022), without relying on complex language skills (McWhirter 2014). Although children's drawings enable an effective exploration of their understanding of COVID‐19 (e.g., Bonoti et al. 2022; Christidou et al. 2021), the DTW has not been used in this context. Younger children tend to use anthropomorphism when drawing microorganisms, a trend that decreases as they grow older and develop a more sophisticated understanding of biology (Byrne 2011). Interestingly, in their exploration of children's understanding of COVID‐19, Labotka and Gelman (2023) found that this trend reversed, where older children anthropomorphised the microbe more than younger children. The DTW methodology is, thus, uniquely positioned to capture in depth children's understanding of COVID‐19.

### 1.3 Present Study

This study is part of a larger multimethod project, including interviews and creative methods, to examine developmental changes in children's understanding of COVID‐19. The study presented in this paper explored children's understanding of COVID‐19 after the pandemic using the DWT method. It aimed (1) to assess the accuracy of children's knowledge as represented in drawings in terms of the dimensions of the CSM, (2) to examine developmental trends within the data and (3) to explore the visual representations children attribute to COVID‐19 in their drawings: illness, public health messaging and microbe.

## 2 Methods

### 2.1 Participants

Seventy children, 5–11 years old, participated in the DWT activity (*M* = 8.5, *SD* = 2.1). Table 1 provides a comprehensive view of the demographic characteristics.

**TABLE 1:** Demographic characteristics.

		Age		Had COVID‐19		Knew someone who had COVID‐19	
Participants	*n*	M	SD	*n*	%	*n*	%
Total	70	8.5	2.1	44	62.9	50	71.4
Younger group	28	6.1	0.9	13	18.6	12	17.1
Older group	42	9.9	1.1	31	44.3	38	54.3
Girls	34	8.2	2.0	18	52.9	24	70.6
Boys	35	8.7	2.2	25	71.4	25	71.4

*Note: n* = 1 participant who marked their gender identity as ‘other’ was not included in gendered statistics. When gender groups are presented above, their information was not included.

### 2.2 Materials

An A4 sheet of white paper and a range of colourful pens, pencils, markers and crayons were provided for the drawing task.

### 2.3 Procedure

Ethical approval was granted by the Informatics Research Ethics Process at the University of Edinburgh and the relevant local authority. Data collection took place across 15 days between November and December 2023. The first author undertook all data collection to ensure consistency, meeting individually with all participants. The study recruited all children attending primary school to ensure just and equitable involvement (Ethical Research Involving Children, n.d.). Informed consent was sought both from parents and children, specifying that the anonymised drawings and quotes of children can be reproduced in publications. All participants were attending the same state‐funded primary school in an area that is part of the least deprived 20% of Scotland, UK. Data collection was carried out during normal school hours. The researcher was introduced to the children in each classroom by the classroom teacher, then individually collected each child whose parents had consented to participate from their classroom. Data collection took place in a separate room on the school premises. During the walk to the study room, informal conversation was struck to build rapport with the child. Upon receiving a child‐friendly participant information sheet and asking any questions, the child could elect to participate in the study; if they did not wish to participate, they were returned to their classroom. Participants' comfort was monitored across the session, and they could withdraw from the study at any time.

The researcher met one participant at a time to capture their distinct concepts and not ideas generated due to a group interview (Einarsdóttir 2007), while also giving the space to less articulate or shy children to express their ideas (Clark 2005). All data were anonymised at the point of collection by assigning a unique identification number. The session with each child included a four‐part data collection process addressing three studies, and it was audio‐recorded. The drawings collected with the DTW activity, presented in this paper, constituted the third part. The session started with demographic questions, then moved to the second part, including a short vignette to describe the COVID‐19 symptoms in a child, followed by an interview. These interview data are presented in a separate paper (in review).

Of the 75 children participating in the interviews, 70 completed the DWT activity, which was the third step of the session and lasted approximately 10 minutes. Children were asked, ‘Would you like to show me what COVID‐19 looks like? You can use both drawings and words’. The DTW activity and instructions were as simple as possible (Carter and Ford 2013), as the aim of the activity was to help elicit their understanding of COVID‐19 and intentionally broad to explore any aspect of their representation; illness, public health messaging and microbe. The five children who elected not to complete a drawing, though, participated in the interviews, quoted reasons such as not knowing what COVID‐19 looks like. During the DWT activity, the researcher remained in the room without prompting for more questions. The children were able to ask questions or speak to the researcher while drawing. Upon completion of the drawing, with or without written text, children were invited to describe their picture to the researcher. If not all parts of the drawing or text were commented on, the researcher prompted the children to provide further explanation to avoid misinterpretations of the children's drawings. Upon each child's agreement, the researcher annotated the drawings based on the verbal explanation of the drawing. This activity ended with the researcher summarising what was in the picture and asking the child if there was anything missed. If needed, further notes were added. This complete visual artefact, comprising the child's original drawing, and accompanying words, and annotations added by the researcher, formed the dataset for the current study and will hereafter be termed the drawing. The drawing was then digitised and handed back to the participant. The session ended with the fourth part: another short interview. These interview data are also presented in a separate paper (in preparation). On completing the session, participants received child‐friendly debriefing, a custom sticker and an ‘Honourary COVID‐19 scientist’ certificate (Ethical Research Involving Children, n.d.).

### 2.4 Analysis

A collaborative analysis was applied to the drawings, taking into consideration the dimensions of difference between the authors (Cornish et al. 2014). Initially, four coders (RM, FM, SZ and JW) familiarised themselves with the drawings and discussed them; In turn, the first three coders conducted the analysis in parallel. The coders met in total eight times for several hours each time to allow for an in‐depth analysis. The analysis of the drawings was twofold; firstly, content analysis (Krippendorff 2019; Weber 1990) was applied to the drawings as a top–down theory‐driven method to assess children's drawings in terms of the dimensions of the CSM. Following, reflexive thematic analysis (Braun and Clarke 2023) was used to explore the key themes depicted in their illustrations of COVID‐19. During analysis, the drawings were separated from the identification numbers to reduce subjective bias. Descriptive statistics of the demographic information were produced using R, while the drawings were initially printed and analysed by hand; subsequently, the dataset and codebook were fully digitised using NVivo 14 (Lumivero 2023). Each analysis is detailed in the following sections.

#### 2.4.1 Content Analysis

Content analysis used a nine‐dimensional coding scheme informed by the CSM. The nine dimensions coded were Identity, Severity, Symptoms, Cause, Timeline (from infection to symptoms and symptoms to recovery), Prevention, Curability and Treatment. For each dimension, children's drawings were assigned a score of 0–3 (0 = *uncodable*, 3 = *high accuracy*). Three coders (RM, FM and SZ) refined the coding scheme repeatedly using a subset of the data to ensure consistency in coding. The complete coding scheme is provided as Table S1. When agreement was reached, FM and SZ applied the coding scheme to the whole dataset, reaching satisfactory intercoder reliability (*κ* = 0.81, 91% agreement); any disagreements were resolved with further discussion. The coding scheme showed good internal consistency (*ω* = 0.73), indicating that the nine dimensions form a coherent scale (Hayes and Coutts 2020). An ‘overall accuracy’ score was also calculated as a mean value of each participant's scores across the nine dimensions. The resulting quantitative data were analysed using R. A nonparametric Wilcoxon rank sum test was used to assess the differences in accuracy between younger (5–7 years old) and older children (8–11 years old).

#### 2.4.2 Reflexive Thematic Analysis

Reflexive thematic analysis was applied to the drawings to capture a more comprehensive view of children's understanding of COVID‐19 without restricting it to the CSM framework. Via extensive iterative discussions, the first three coders (RM, FM and SZ) analysed the data, which enhanced the richness, nuance and rigour of the analysis as multiple perspectives of the drawings were incorporated (Braun and Clarke 2019; Wiltshire and Ronkainen 2021). Initial codes were generated, which were then organised into potential themes, subsequently refined into definitive themes and subthemes, from which a thematic map of the data was produced (see Figure 1).

**FIGURE 1:**
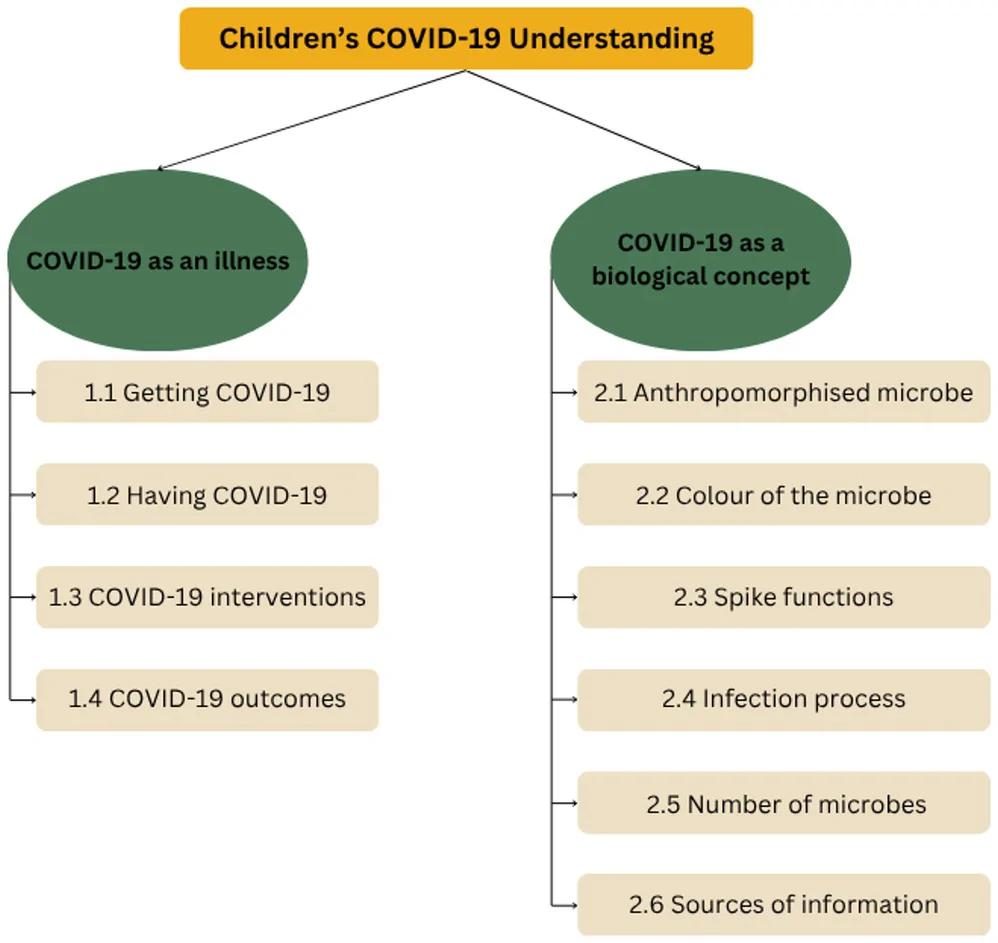
The thematic map.

### 2.5 Positionality and Reflexivity Statement

This study was grounded in the philosophical position of Critical Realism, paired with an Interpretivist epistemology. Reflexivity is an important ethical consideration when conducting research with children (Graham et al. 2015); thus, RM kept a reflexive journal during data collection. The authors acknowledge that their assumptions, values, attitudes, beliefs, experiences and expertise around children and COVID‐19 can influence their interpretation of the data. Children are recognised as social actors, thus capable of providing informed consent, having the right to comment on topics that concern them, and experts in their own experiences. The COVID‐19 pandemic is accepted as a pivotal moment of universal scope, notwithstanding differences in geographical and political responses. All authors have had personal or professional experience with COVID‐19 and the pandemic. The authors' positionality was considered and extensively discussed during the data analysis.

## 3 Results

### 3.1 Content Analysis

Participants' mean overall accuracy across all nine dimensions of the CSM was compared between the younger and older groups. Table 2 presents the Wilcoxon test results for all dimensions. Older children had statistically significantly higher (*W* = 202.5, *p* < 0.001) median overall accuracy in their understanding of COVID‐19 within the CSM (median = 0.44) than younger children (median = 0.11).

**TABLE 2:** Wilcoxon test results for all dimensions.

Dimension	Age group	*n*	Mdn	IQR	*W*	*p*	*r*
Overall Accuracy	Younger	28	0.11	0.25	202.5	< 0.001***	0.66
	Older	42	0.44	0.33			
Identity	Younger	28	1	1	360	< 0.001***	0.39
	Older	42	1	0			
Severity	Younger	28	0	0	462	0.01**	0.21
	Older	42	0	0			
Cause	Younger	28	0	0	235	< 0.001***	0.60
	Older	42	2	3			
Symptoms	Younger	28	0	0.25	518	0.303	0.12
	Older	42	0	1			
Prevention	Younger	28	0	0	550.5	0.533	0.06
	Older	42	0	0			
Timeline to symptoms	Younger	28	0	0	560	0.253	0.05
	Older	42	0	0			
Curability	Younger	28	0	0	580	0.798	0.01
	Older	42	0	0			
Treatment	Younger	28	0	0	551.5	0.477	0.06
	Older	42	0	0			
Timeline to recovery	Younger	28	0	0	528	0.17	0.10
	Older	42	0	0			

*Note: W* = Wilcoxon test statistic; *p* = *p*‐value; *r* = effect size; Mdn = median; IQR = interquartile range.

Investigation of the individual dimensions of the CSM revealed that the Identity (*W* = 360, *p* < 0.05), Severity (*W* = 462, *p* < 0.05) and Cause (*W* = 235, *p* < 0.001) dimensions significantly increased in accuracy between younger and older children. However, there were no changes in Symptoms, Prevention, Timeline to symptoms, Curability, Treatment or Timeline to recovery, which were consistently low across age (See Table 2).

### 3.2 Reflexive Thematic Analysis

Two themes surrounding children's understanding of COVID‐19 were constructed from the data: (1) COVID‐19 as an illness and (2) COVID‐19 as a biological concept (see Figure 1). These themes and subthemes demonstrate that children's understanding of COVID‐19 is multifaceted, encompassing both the concept of the illness and its biological aspects. These are discussed below in detail with key indicative quotes and drawings.

### 3.3 Theme 1—COVID‐19 as an Illness

Theme 1 encompasses children's understanding of COVID‐19 as an illness and consists of four subthemes that align with some dimensions of the CSM. Participants illustrated the process of getting ill with COVID‐19 (3.3.1), what it is to be ill (3.3.2), what a patient can do when ill (3.3.3) and what the potential outcomes are (3.3.4). This theme is closest aligned with the CSM; however, it does not precisely follow it. Drawings falling under this theme mainly included images of humans, though illustrations of the microbe were present. Although there are developmental patterns within the subthemes, the drawings are from across the age range.

#### 3.3.1 Getting COVID‐19

This subtheme covers children's understanding of modes of transmission of COVID‐19 and infection prevention methods (see Figure 2). Drawings included in this subtheme were produced by participants aged 6–11, with a mean age of 9.

**FIGURE 2:**
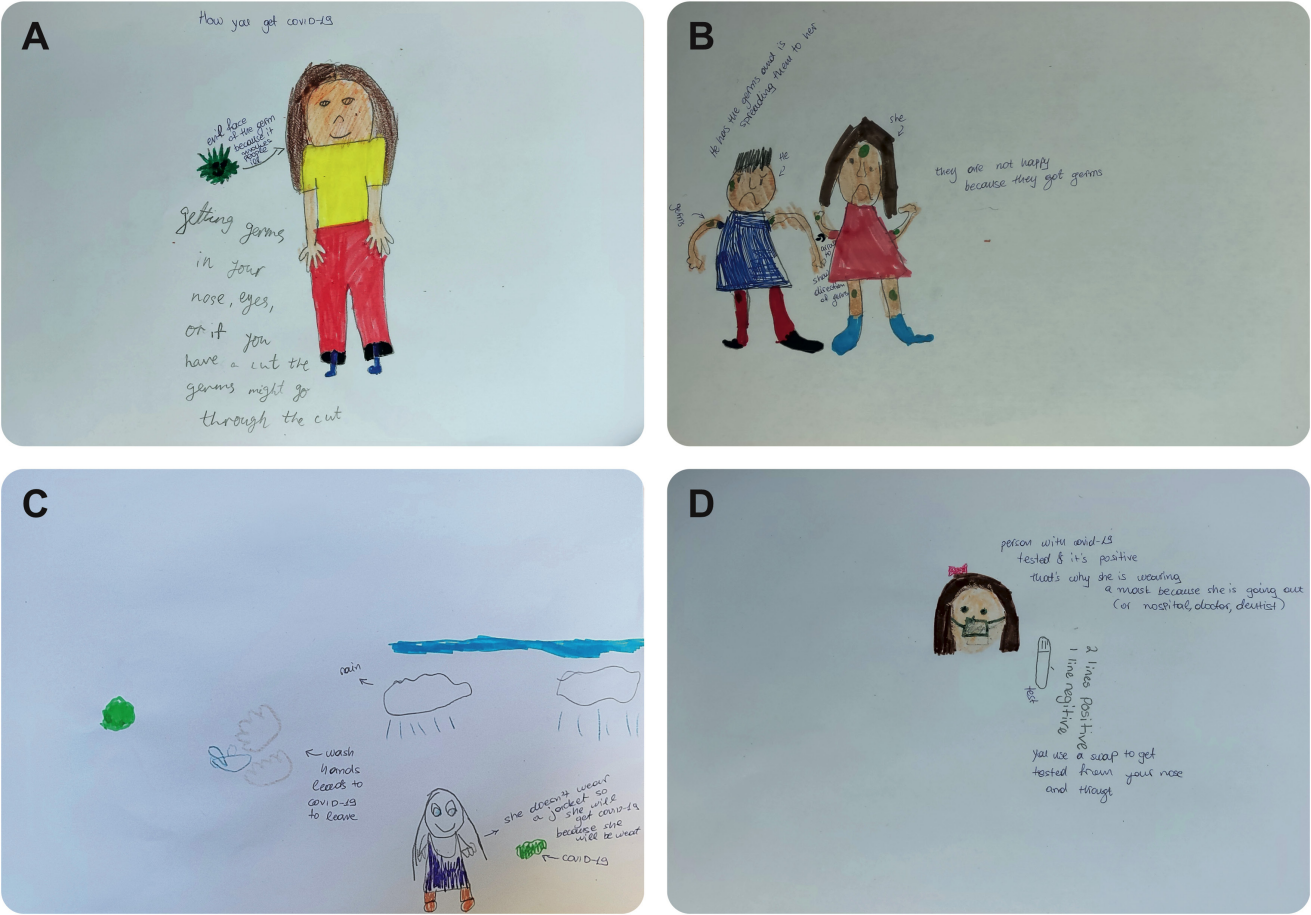
Composite of participant drawings representing the subtheme of ‘Getting COVID‐19’. (A) Participant 46 focuses on mechanisms of contagion. (B) Participant 31 focuses on the transmission process. (C) Participant 16 focuses on handwashing. (D) Participant 71 focuses on wearing a mask.

Participants portrayed and talked about mechanisms of contagion, such as saliva, nose and eyes, hands and dirt. Children's depictions also point towards a conceptualisation of the transmission process, where they represented their understanding of the virus spread. They also addressed prevention strategies, including handwashing, wearing a mask and covering one's mouth when coughing. Interestingly, children did not illustrate vaccination against COVID‐19 as a prevention strategy.

#### 3.3.2 Having COVID‐19

This subtheme encompasses children's understanding of what it means for one to be ill with COVID‐19 (see Figure 3). They illustrated physiological, psychological and social aspects of the illness. These drawings were produced by participants aged 5–11, with a mean age of 8.3. Notably, this subtheme has the lowest mean age across the subthemes.

**FIGURE 3:**
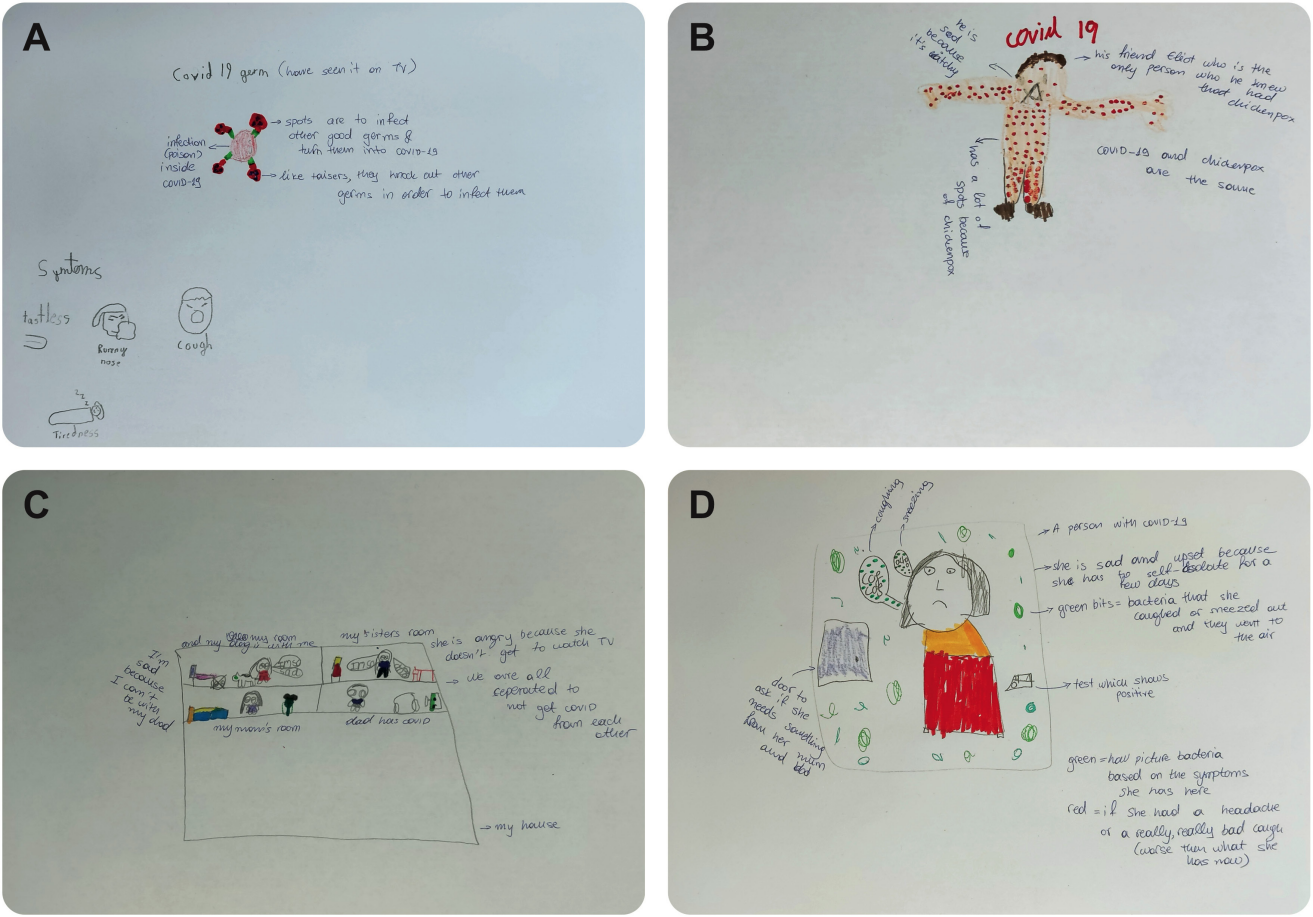
Composite of participant drawings representing the subtheme of ‘Having COVID‐19’. (A) Participant 55 focuses on the COVID‐19 symptoms. (B) Participant 28 focuses on symptoms of a different disease. (C) Participant 34 focuses on emotions. (D) Participant 69 focuses on self‐isolation.

Physiological aspects included the COVID‐19 symptomatology. Some children showed a very accurate understanding of what the symptoms are, while others misattributed symptoms of other illnesses familiar to them, for example, chickenpox. Several drawings presented psychological aspects of having COVID‐19. The most common emotions expressed were sadness, followed by anger and loneliness. No participant mentioned feelings of fear or any positive emotions towards having COVID‐19, for example, being happy to stay home from school. Social aspects of having COVID‐19 were also represented. Drawings showed people either being alone, for example, self‐isolating, or being with others, who were taking care of them while ill or where about to become infected from them.

#### 3.3.3 COVID‐19 Interventions

This subtheme comprises children's understanding of interventions for COVID‐19 (see Figure 4). Interventions were considered both the condition's assessment and treatment. This subtheme had a mean age of 9.3 years, with older children mentioning condition assessments, while younger children mainly mentioned treatments.

**FIGURE 4:**
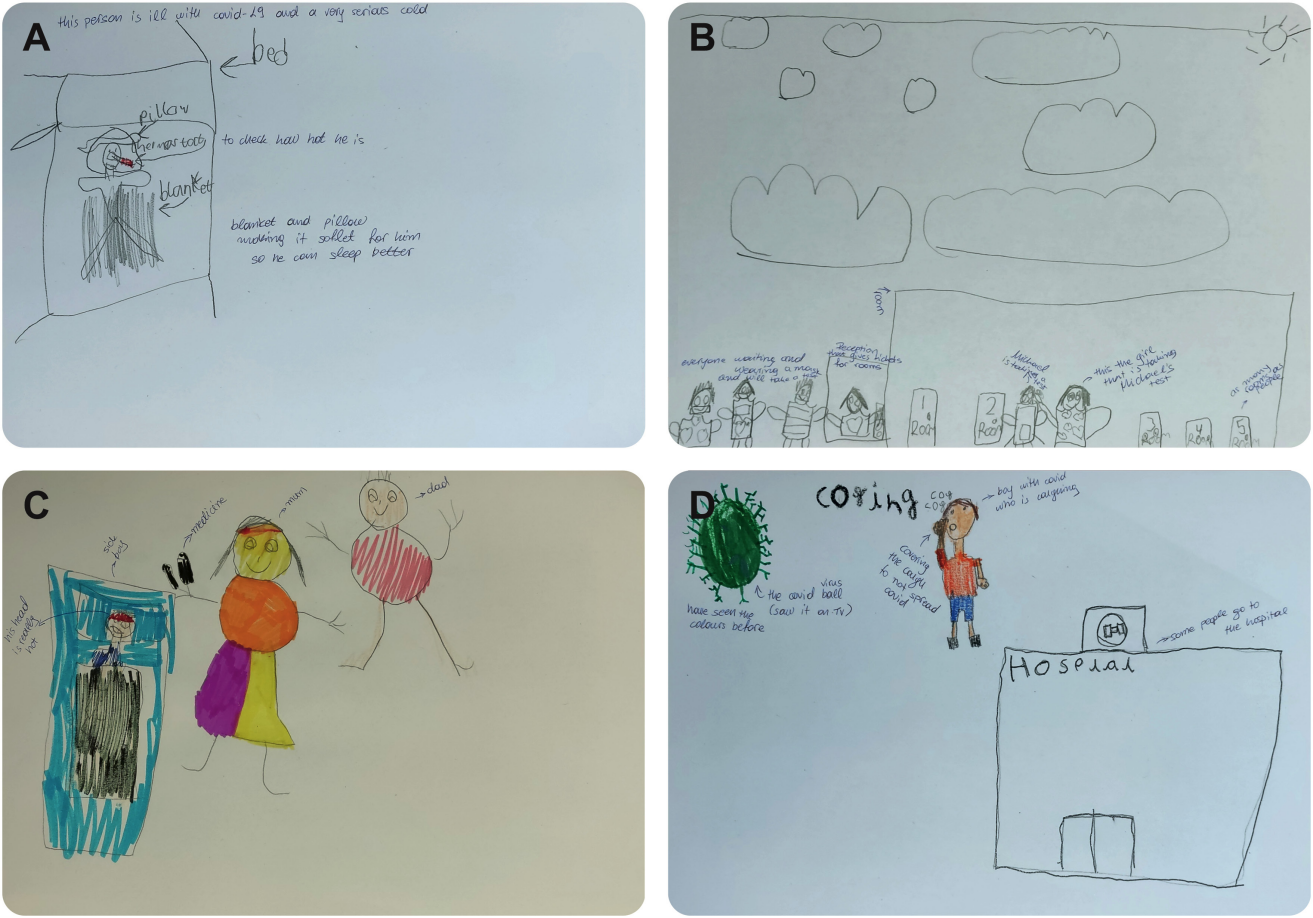
Composite of participant drawings representing the subtheme of ‘COVID‐19 interventions’. (A) Participant 48 focuses on the use of a thermometer to assess symptoms. (B) Participant 23 focuses on a testing centre. (C) Participant 13 focuses on a generic medicine. (D) Participant 38 focuses on the need to go to the hospital.

Drawings presenting an assessment for COVID‐19 referenced both at‐home and professional symptom evaluation and diagnostic testing. For at‐home assessment, mainly thermometers were used to measure fever, or Rapid Lateral Flow Antigen Tests were presented as a screening tool for COVID‐19. Professional evaluations included pictures of PCR (Polymerase Chain Reaction) tests at testing centres. Drawings also illustrated treatments available for COVID‐19, with the majority focused on at‐home symptom relief and self‐care. Children emphasised generic, unnamed ‘medicine/medications/pills’ to treat symptoms, at‐home remedies and staying in bed to rest. Some drawings referenced medical professionals or the need to go to the hospital, but none showed treatments in a hospital setting.

#### 3.3.4 COVID‐19 Outcomes

The final subtheme illustrated children's understanding of the outcomes of a COVID‐19 infection (see Figure 5). The outcomes presented a binary of either complete recovery or death, with a relatively even split between the two. Children did not present a great level of detail about either of the outcomes in their drawings and more often used words to describe it: ‘can be life threatening’ (P63). The outcomes were attributed to different factors, with the majority connecting them to different treatments (see subtheme 1.3) and one participant to the year of infection. Drawings containing this subtheme were created by children aged 8–11, with a mean age of 10.1, which is the highest mean age across the subthemes.

**FIGURE 5:**
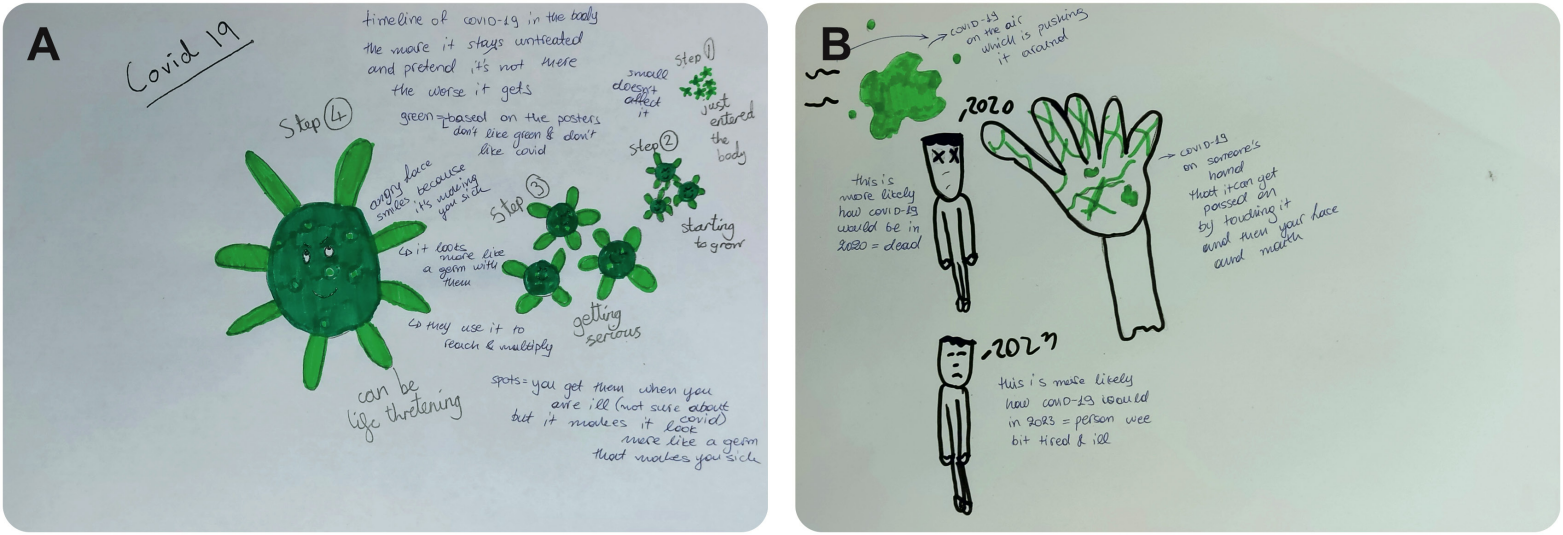
Composite of participant drawings representing the subtheme of ‘COVID‐19 outcomes’. (A) Participant 63 focuses on the potential of death. (B) Participant 39 focuses on connecting the outcome to the year of infection.

### 3.4 Theme 2—COVID‐19 as a Biological Entity

Theme 2 encompassed the largest segment of the analysis. It captured children's biological conceptualisations of COVID‐19 as a biological entity and is organised into six subthemes. Children attributed human characteristics and behaviours to the microbe (3.4.1), used colour to convey information about it (3.4.2), ascribed different functions to the microbe's spikes (3.4.3), visualised the infection process (3.4.4), illustrated a single or multiple microbes to convey this information (3.4.5) and cited their sources of information (3.4.6). This theme illustrates children's attempts to render the invisible biological virus into a visible form. In contrast with Theme 1, drawings captured by Theme 2 mainly include, or focus only on, illustrations of the microbe rather than portraying people. These drawings were primarily drawn by older children, suggesting a developmental trend.

#### 3.4.1 Anthropomorphised Microbe

This subtheme encompassed children's anthropomorphism of the microbe in a variety of ways, such as human‐like physical characteristics, emotions, social dynamics and morality (see Figure 6). This subtheme was mainly evident in the drawings of older children, with a mean age of 9 and a range of 6–11 years. Interestingly, younger children (*M*_age_ = 7.3) tended to draw microbes engaging in social dynamics, while older children (*M*_age_ = 9.9) depicted children acting immorally (e.g., P47).

**FIGURE 6:**
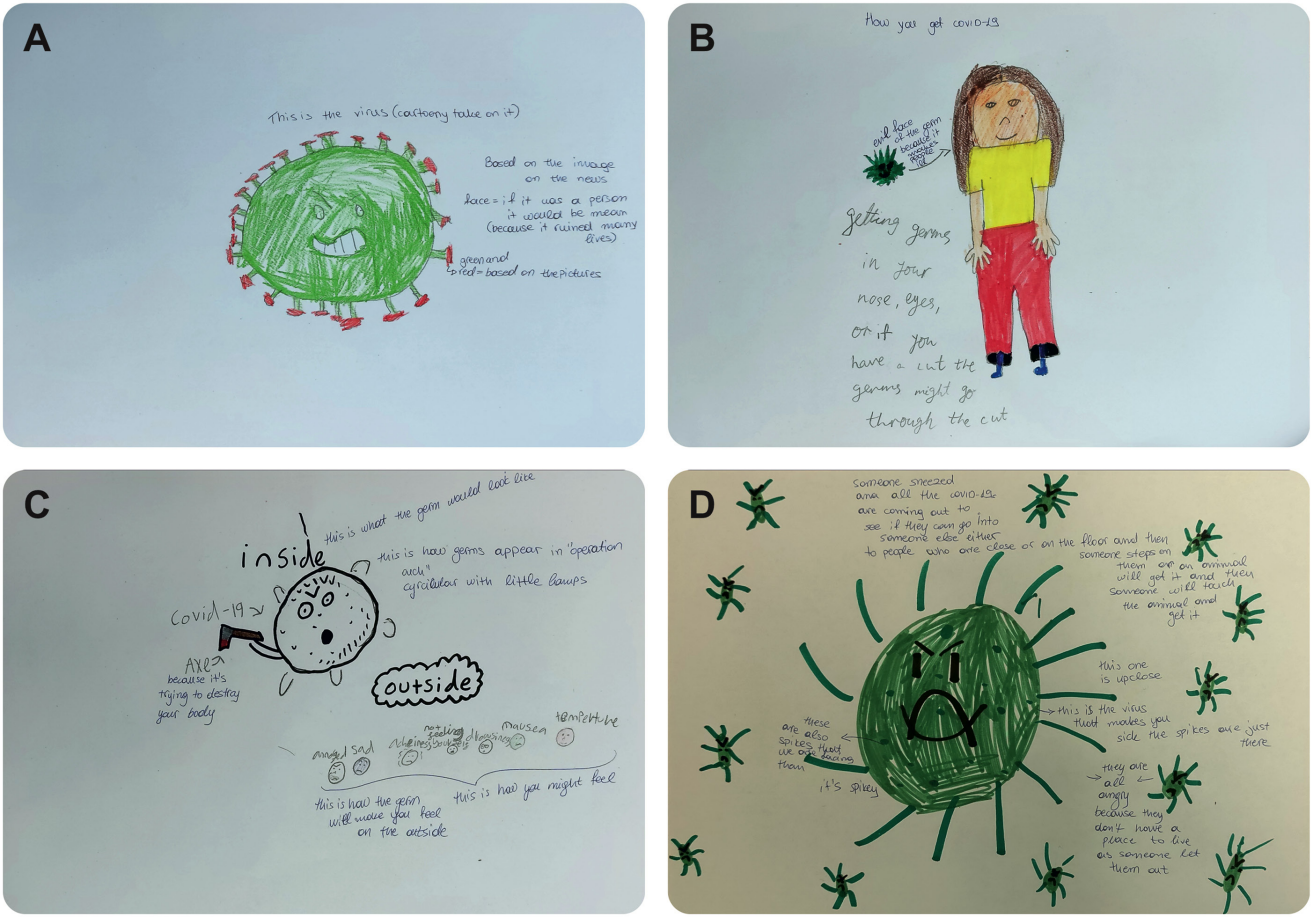
Composite of participant drawings representing the subtheme of ‘anthropomorphised microbe’. (A) Participant 72 focuses on a cartoony take of the virus and attributing moral characteristics to it. (B) Participant 44 focuses on geometric figures in the virus's microscopic imagery. (C) Participant 47 focuses on the use of a weapon from the virus. (D) Participant 35 focuses on the emotions of the virus.

Children, when drawing the microbe, gave it human‐like physical characteristics, such as a face with eyes and a mouth. These characteristics added functionality to the virus: ‘It has eyes, so it can move’ (P26). The anthropomorphistic choices could be attributed to the visualisations of microorganisms in children's media (Ganea et al. 2014), ‘this is the virus; a cartoony take on it’ (P72); however, P44 provided a different explanation: ‘it has black spots that you see in a microscope, and sometimes they form/look like a face’, denoting a way of meaning‐making of the geometric figures in the microscopic imagery. A moral code and intention were also attributed to the microbe, deeming it ‘not nice’ (P41), or clutching weapons, such as an axe or tasers. Similar to the physical characteristics, the microbe's use of weapons was explained as serving a functional role, either ‘knocking out other germs in order to infect them’ (P55) or ‘trying to destroy your body’ (P47), notwithstanding the depiction of a weapon as a metaphorical symbol of the immune system attacking the virus (P56).

Drawings were also produced, in which the microbe was pictured exhibiting human‐like emotions. The virus was portrayed as *‘angry’* even when smiling. This anger was attributed to its morality ‘if it was a person it would be mean because it ruined many lives’ (P72) or a change to the virus's status quo, e.g., as a response ‘when you spray something (sanitiser) on its face’ (P41) or ‘they don't have a place to live as someone let them out (someone sneezed)’ (P35). Children also depicted social dynamics within clusters of microbes, where one or more would be the leader, and others would follow them: ‘king and queen germs’ (P15). These choices could be seen as a way in which children tried to understand the effect that COVID‐19 had on people's lives.

#### 3.4.2 Colour of the Microbe

Children, across the age range, used colour in their drawings as an informative marker about the microbe, rather than in an affective way (see Figure 7). Both hue and saturation played a role in children's narratives, with some participants attributing colours to denote the virus's physiology, assigning a different colour to the body and the spikes, or different colours to different symptoms. Some also based their choice of colours on illustrations of the virus in the media or the ‘sick emoji’. Others mentioned that they guessed the colours, while mirroring the colours used in a lot of health messaging. The most common hues used were green, red and grey. Many participants chose green as the colour that represents illness, red as ‘it is covered in blood by travelling through the bloodstream, otherwise green’ (P66), suggesting the virus is bloodborne and when grey was used, it differentiated the body from the red spikes. Saturation was mainly used to highlight the effectiveness of the different molecules, with darker‐coloured ones being stronger or more infectious compared to the lighter ones.

**FIGURE 7:**
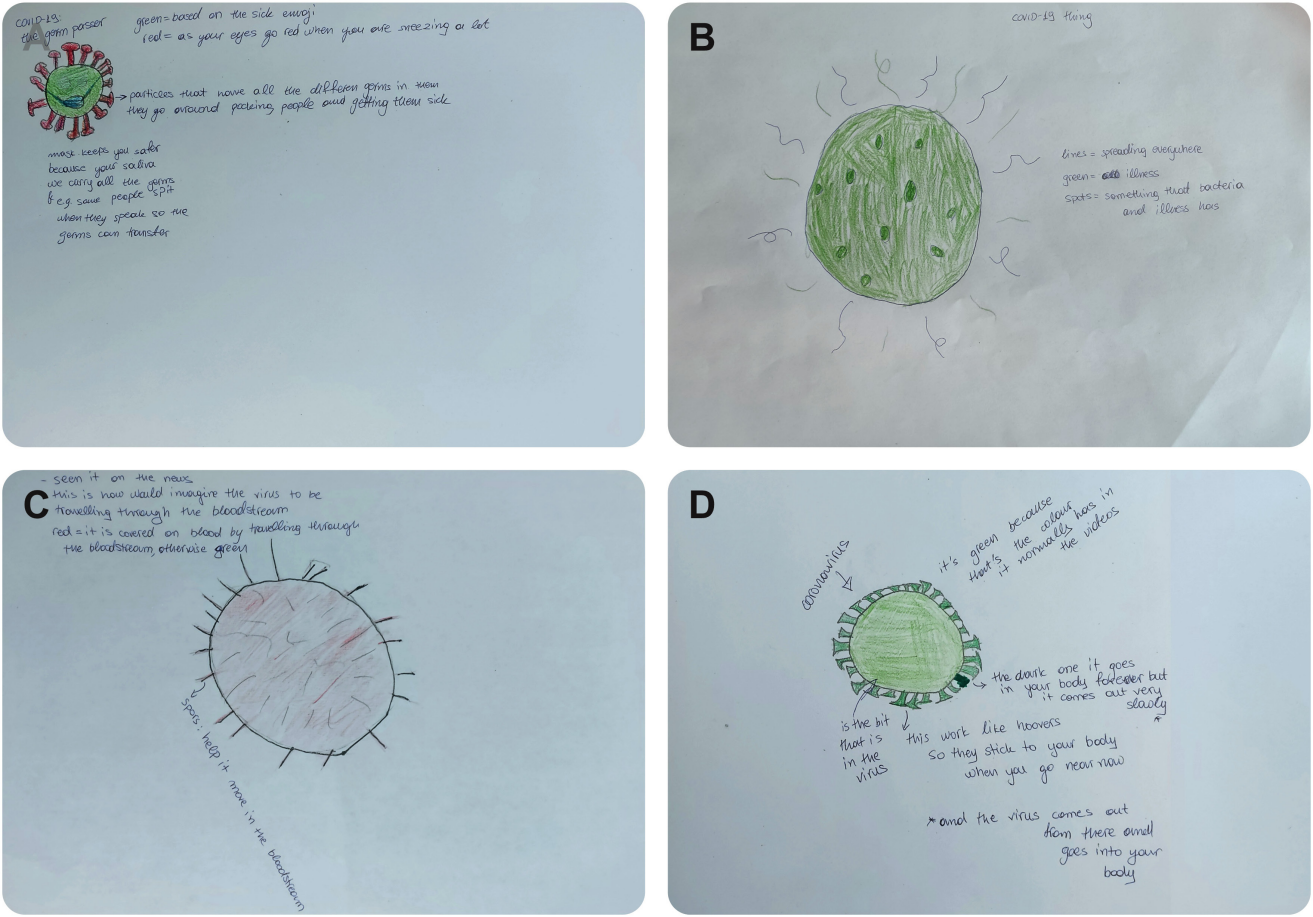
Composite of participant drawings representing the subtheme of ‘Colour of the microbe’. (A) Participant 73 connects green to the sick emoji. (B) Participant 57 connects green to the colour of illness. (C) Participant 66 focuses on red to signify the disease as bloodborne. (D) Participant 33 focuses on colour saturation to signify effectiveness.

#### 3.4.3 Spike Functions

The microbe was most often illustrated as having spikes, and the participants provided elaborate explanations on their functionality (see Figure 8). This subtheme has the highest mean age at 11 years old, indicating a potential developmental trend. Children explained that the spikes secure the cell's structural integrity, holding the illness in place; they act as defence against other cells (e.g., white blood cells [P59] or an infection mechanism to other cells, accurately recognising the role of spike proteins on the SARS‐CoV‐2 virus). Other explanations given were spikes enabling purposeful navigation towards specific targets within the body, mobility, where spikes help soar through the air or attachment to objects, with several participants describing a ‘hoover’ or suction effect.

**FIGURE 8:**
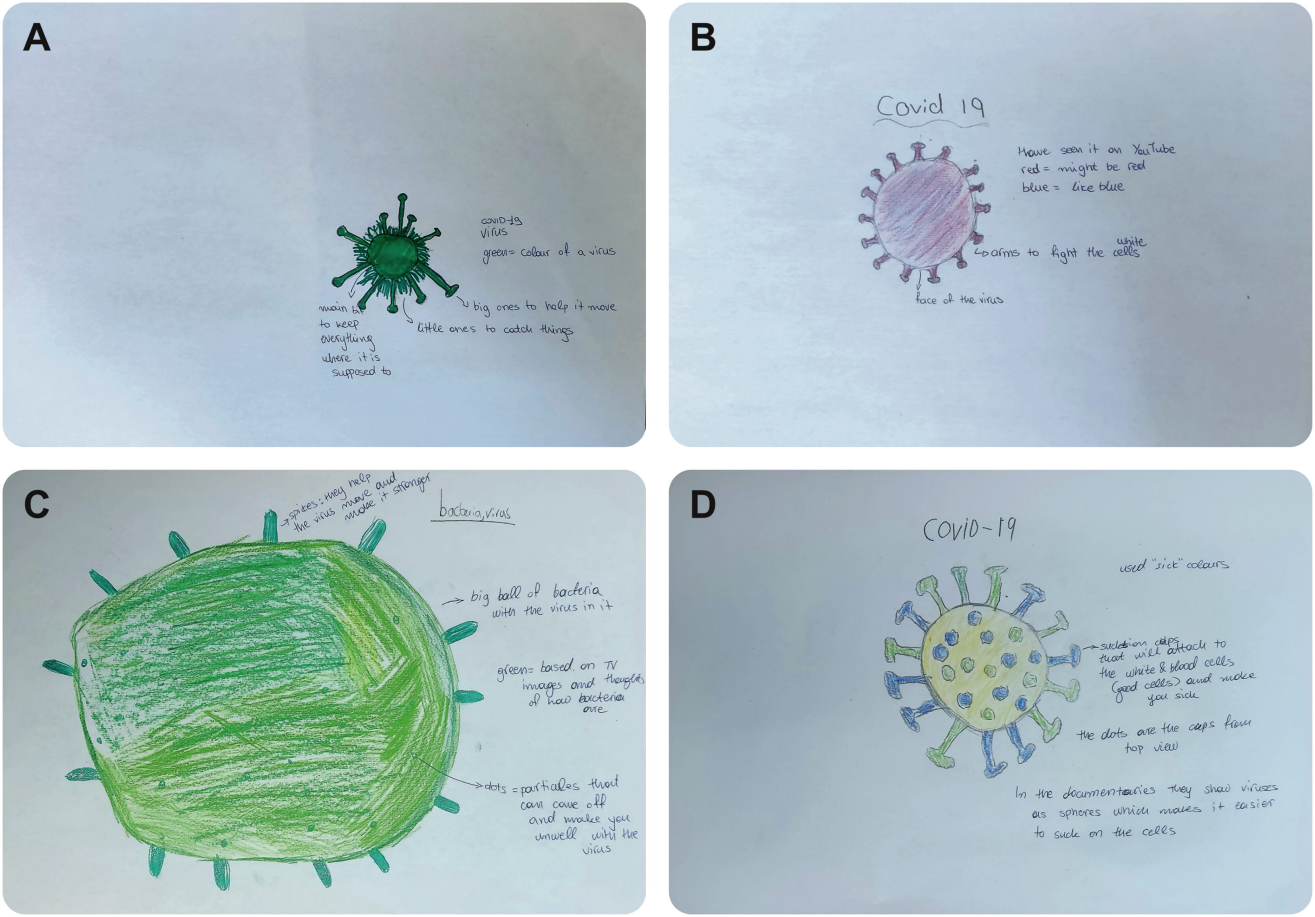
Composite of participant drawings representing the subtheme of ‘Spike functions’. (A) Participant 61 focuses on multiple functions, for example, structural integrity. (B) Participant 59 focuses on defence against white blood cells. (C) Participant 68 focuses on aiding movement. (D) Participant 51 focuses on suction.

#### 3.4.4 Infection Process

Participants illustrated their conceptualisation of the COVID‐19 infection process (see Figure 9). This subtheme had a mean age of 9, with an age range of 6–11 years old. They presented the infection process as the virus multiplying within the body (P63) or ‘taking over the healthy cells […] make unhealthy cells’ (P43). They highlighted the role of the immune system in the process, mentioning white blood cells and antibodies ‘Once you have had covid 19 your body will remember it […], each time you get it, the quicker it will go away’ (P56). This demonstrated a quite sophisticated understanding of how infections take place in the human body.

**FIGURE 9:**
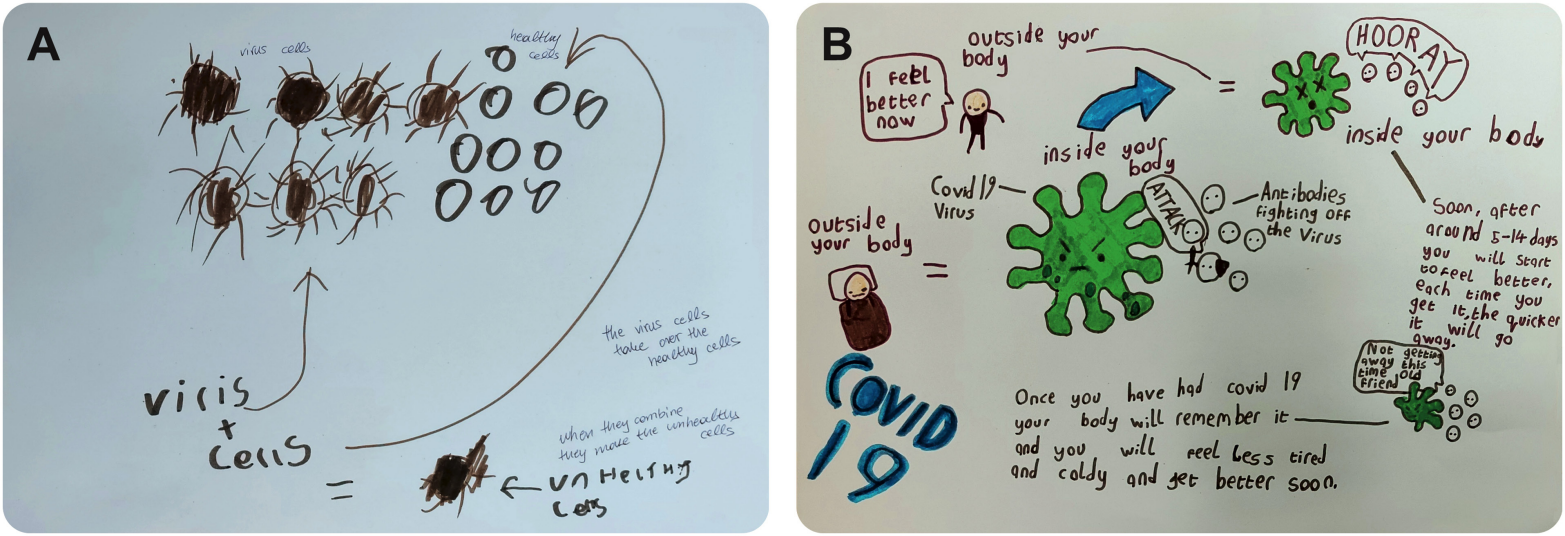
Composite of participant drawings representing the subtheme of ‘Infection process’. (A) Participant 43 focuses on the virus taking over the healthy cells. (B) Participant 56 focuses on the infection process as a whole.

#### 3.4.5 Number of Microbes

Children drew either a single or multiple microbes to represent COVID‐19. Participants who drew individual microbes tended to be slightly older than those who drew groups of microbes (M_age_ = 10 and M_age_ = 9, respectively). Interestingly, this subtheme encompassed drawings across the theme and was not limited to describing the number of microorganisms needed for an infection to occur or to denote the severity of the disease. Children focused more on the physiology of the microbe when designing only one compared to several.

#### 3.4.6 Sources of Information

Children cited their sources of information in shaping their understanding of what the COVID‐19 virus looks like. They mainly referenced images and videos with older children, citing the latter. No child mentioned their parents or teachers as direct sources of information. Participants who cited videos mentioned the news as their main source of information. Interestingly, they had produced visualisations that resembled the one in the BBC imagery. Others had mentioned specific children's TV programmes, including *Operation Ouch!* on CBBC (i.e., a long‐running series on the BBC presented by three medical doctors aimed to educate children about medicine and biology). This underscores the influence of the visualisations used by mass media in health communications.

## 4 Discussion

This DWT study explored how primary school children understand COVID‐19 after the pandemic. Employing a dual analysis methodology, where content and reflexive thematic analyses were applied to the data, cultivated a rich ground to investigate children's COVID‐19 concepts. The CSM provided a foundational framework to identify clear developmental trends in their overall understanding and knowledge gaps across the different dimensions. Key themes illustrated in their drawings of COVID‐19 were also found, highlighting COVID‐19 conceptualisation as multifaceted and sophisticated, ranging from an illness to a biological concept.

Content analysis acted as a deductive approach to view the data through a theoretical lens, demonstrating a clear developmental progression in children's understanding of COVID‐19. Children's drawings reflected the features of the CSM but notwithstanding variation across age and dimensions. Although older children showed higher overall accuracy in their understanding, this was particularly pronounced in the dimensions of Identity, Severity and Cause. Across all ages, the dimensions of Timeline, Curability and Treatment were consistently underdeveloped. This observed asymmetry suggests that some aspects of the COVID‐19 concept may be more developmentally sensitive, highlighting knowledge gaps and the need for focused educational interventions for these areas.

Reflexive thematic analysis allowed for an inductive approach to explore the richness of the data, leading to deepened insights about the children's visual representations attributed to COVID‐19 in their drawings. COVID‐19 was depicted as a complex concept with biological, psychological and social representations. A unique finding of this study, not previously discussed in the literature, is the functions that children attributed to the virus's spikes. It provided a window into the way children construct scientific meaning and indicated a form of naïve biology (Hatano and Inagaki 1994), as they assigned agency and biological purpose to microbes. Similar to earlier studies (Bonoti et al. 2022; Byrne 2011), children older than 7 years old more often drew one microbe with a great level of detail, presenting a more scientific version of the virus.

Interestingly, compared to traditional Piagetian perspectives on children's illness concepts (e.g., Bibace and Walsh 1980), children anthropomorphised COVID‐19 across all ages, with older children being more likely to do so. This finding aligns with Labotka and Gelman (2023) highlighting a potentially unique characteristic of the children's COVID‐19 conceptualisation. Importantly, anthropomorphism appeared in different ways, one being perceptual (e.g., a child imagining a face based on the lines on a view of the virus from a microscope), supporting Airenti's (2018) argument that anthropomorphism is a basic human attitude and not specific to children. Children also attributed an affective state and moral disposition to the virus. This could be attributed to the pandemic's moral undertones in the UK public health messaging, highlighting the importance of media for health communication. Another potential driver may be effectance motivation to create a sense of control (Waytz et al. 2010). As COVID‐19 was an invisible, unpredictable health threat, older children may have used anthropomorphism to help explain this complex stimulus, reduce uncertainty and increase confidence in the predictions of how it would act in the future (Epley et al. 2007).

Illness‐related emotions were presented, though supporting earlier research that children attach emotional load to illness concepts (Strömmer et al. 2022; Thompson et al. 2021), but with a noticeable absence of fear and positive emotions about COVID‐19, which has been found previously (Bonoti et al. 2022). The lack of these particular emotions could potentially be attributed to the time that has passed since COVID‐19 was a pandemic.

Children did not mention their parents or teachers as their sources of information, rather mentioning the news or imagery, a striking difference from Bray, Carter, et al. (2021)'s international study during the first wave of COVID‐19, which named them as children's only information sources. This highlights a potential shift in children's health information acquisition during and after the pandemic, towards more child‐friendly information, potentially due to the change in familiar and societal health literacy assumptions about what children should know after the end of the pandemic.

## 5 Strengths and Limitations

This study benefits from several significant strengths. The collection of 70 drawings constitutes an exceptionally large dataset for a DWT study. Furthermore, the balanced gender sample mitigates potential gender‐based biases in conceptualisation, thereby strengthening the credibility of the results. Methodologically, using the DWT as a data collection method and the CSM as a theoretical framework, provided a rich dataset of children's COVID‐19 understanding. Lastly, the dual analytical lenses of content and reflexive thematic analyses provided a robust depth and breadth of analysis of children's COVID‐19 understandings, generating a range of novel findings.

The limitations that often come with cross‐sectional studies are also acknowledged. Causal inferences about the effect of development on the evolution of children's understanding of COVID‐19 are limited. Future research could employ a longitudinal design to investigate the way COVID‐19 concepts develop with age. Similarly, although a large sample was recruited, all participants were attending the same primary school, which could hinder the transferability of the findings. Further research could include participants from diverse sociodemographic backgrounds. Finally, while all data were collected by the same researcher, enhancing consistency, this also risks introducing unintended bias in the data. This was mitigated by the reflexivity of the researcher. However, future research could also consider employing multiple researchers for the data collection, notwithstanding the increased resource intensity of this approach.

## 6 Implications

As future pandemics are likely (Marani et al. 2021) and infectious diseases are omnipresent in the community, the results of this study have significant, actionable implications for child‐friendly health communication and biology education. The observed uneven understanding of the CSM dimensions highlights specific areas that educational interventions should target, that is, timeline, curability and treatment. Furthermore, the prevalence of the anthropomorphised microbe in children's drawings of COVID‐19 creates a strong case for its use in the visualisations designed for communicating effectively child‐friendly information about illnesses. Rather than avoiding it, campaigns should leverage anthropomorphism as a basic human attitude for mean‐making. Ultimately, these findings highlight the importance of visual media in the dissemination of health information to this age group. Finally, an important methodological consideration for future research is the demonstrated efficacy of the DTW method in school settings. It is recommended that future research using this method in schools adopts an open‐ended approach, posing a broad question to children and allowing them ample time to share their insights through both drawings and verbal comments. Such an approach ensures that children's voices are heard and the data collected is comprehensive and nuanced. Within a school context, this process requires that the researcher builds a strong relationship and rapport with the school staff, develops a thorough understanding of the school culture and respects the school's schedule and timings to ensure smooth and consistent participation.

## 7 Conclusion

This study showed that COVID‐19 is understood as a multifaceted concept by primary school children in the postpandemic world. Clear developmental trends were identified in children's drawings. Older children show higher accuracy in their conceptualisation of the Identity, Severity and Cause dimensions of the CSM. Regardless of age, knowledge gaps were identified in the Timeline and Curability dimensions, highlighting that the CSM dimensions are understood unevenly. Furthermore, children's drawings painted a clear picture of their COVID‐19 understanding, expanding beyond the CSM dimensions. COVID‐19 encapsulates both the concept of the illness and its biological entity, including anthropomorphism, morality, naïve biology and social responsibility. These results hold broader implications in the postpandemic world for the design and dissemination of developmentally appropriate child‐specific health information campaigns and biology education in the primary school years.

## Author Contributions

**Rea Evangelia Michalopoulou:** writing – original draft, conceptualization, investigation, funding acquisition, methodology, data curation, project administration, formal analysis, supervision, writing – review and editing, resources, visualization, validation. **Freya Murray:** writing – original draft, formal analysis, writing – review and editing. **Shuran Zhang:** writing – original draft, formal analysis, writing – review and editing. **Benjamin Bach:** writing – original draft, conceptualization, supervision, writing – review and editing. **Panagiotis Kourtesis:** writing – original draft, conceptualization, supervision, writing – review and editing. **Joanne M. Williams:** writing – original draft, writing – review and editing, supervision, conceptualization, formal analysis.

## Funding

Rea Evangelia Michalopoulou acknowledges the support of the Onassis Foundation through the Onassis Scholarships' Program—Scholarship ID: F ZS 038‐1/2022‐2023.

## Ethics Statement

Ethical approval was granted by the Informatics Research Ethics Process (RT #7453) at the University of Edinburgh and the City of Edinburgh Council, which is the relevant local authority.

## Conflicts of interest

The authors declare no conflicts of interest.

## Supporting information


**Table S1:** The complete coding scheme used for the content analysis.

## Data Availability

The data that support the findings of this study are not available to share due to ethical restrictions.
