# Supplementary material for: What Does COVID‐19 Look Like? A ‘Draw, Write and Tell’ Exploration of Children's Postpandemic Understanding of COVID‐19
Source: Child Care Health Dev. 2026 Nov 6;52(6):e70374. doi: 10.1111/cch.70374 (PMC13743276; doi:10.1111/cch.70374)
Supplement: Supplementary file 1 [file CCH-52-e70374-s001.docx]

**Table.**

The complete coding scheme used for the content analysis

| Dimensions | Score | Level | Description |
| --- | --- | --- | --- |
| Identity | 0 | DK/ Un-codable |  |
|  | 1 | Low Accuracy | Recognition of the disease (or pandemic) and/ or show limited understanding of contagion or viral mechanism or microbiological understanding (e.g. by a drawing of the microbe) |
|  | 2 | Mid Accuracy | Recognition of the disease (or pandemic) and show an understanding of contagion and viral mechanism |
|  | 3 | High Accuracy | Show an understanding it is a viral contagious illness (from person to person) |
| Severity | 0 | DK/ Un-codable |  |
|  | 1 | Low Accuracy | Limited understanding of a spectrum of minor illness to hospitalisation to death and no explanation |
|  | 2 | Mid Accuracy | Understanding of a spectrum of minor illness to hospitalisation to death with no explanation |
|  | 3 | High Accuracy | Understanding a spectrum of minor illness to hospitalisation to death with some explanation on the why |
| Symptoms | 0 | DK/ Un-codable |  |
|  | 1 | Low Accuracy | 1-3/12 symptoms |
|  | 2 | Mid Accuracy | 4-6 symptoms |
|  | 3 | High Accuracy | 7-12 symptoms |
| Causes | 0 | DK/ Un-codable |  |
|  | 1 | Low Accuracy | Simple understanding of contagion and no understanding of transmission or virus (no mention of vehicle for transmission) |
|  | 2 | Mid Accuracy | Understanding of contagion, some understanding of transmission, no mention of virus (but use words such as germs, things, particles, bacteria – a vehicle for transmission) |
|  | 3 | High Accuracy | Understanding of contagion, and transmission (explicit mention of the virus) |
| Prevention | 0 | DK/ Un-codable |  |
|  | 1 | Low Accuracy | 1-2 methods |
|  | 2 | Mid Accuracy | 3-4 methods |
|  | 3 | High Accuracy | 5-6 methods |
| Timeline 1  (Infection to symptoms) | 0 | DK/ Un-codable |  |
|  | 1 | Low Accuracy | Little understanding of the time lag or mention of an extreme time lag |
|  | 2 | Mid Accuracy | Understanding of time lag |
|  | 3 | High Accuracy | Understanding time lag with accurate and specific examples |
| Curability | 0 | DK/ Un-codable |  |
|  | 1 | Low Accuracy | Statement of outcome (with incorrect explanation) - understanding that it is curable, no explanation or incorrect explanation |
|  | 2 | Mid Accuracy | Statement of outcome with some explanation – understanding that it is curable with basic explanation |
|  | 3 | High Accuracy | Statement of outcome with full explanation (they mention long covid and death) |
| Treatment | 0 | DK/ Un-codable |  |
|  | 1 | Low Accuracy | Limited understanding of range of treatments (e.g., giving one example of basic home treatment) |
|  | 2 | Mid Accuracy | Basic health treatments of symptoms with relevant examples |
|  | 3 | High Accuracy | Spectrum of basic treatment to doctor to hospitalisation/ death |
| Timeline 2 (Symptoms to recovery) | 0 | DK/ Un-codable |  |
|  | 1 | Low Accuracy | Understanding it takes some time, no understanding of spectrum of recovery |
|  | 2 | Mid Accuracy | Understanding timeframe and spectrum of recovery (no mention of long covid) |
|  | 3 | High Accuracy | Understanding of timeframe and a spectrum of recovery (mention long covid) |
